# The Effects of Various Parameters of the Microwave-Assisted Solvothermal Synthesis on the Specific Surface Area and Catalytic Performance of MgF_2_ Nanoparticles

**DOI:** 10.3390/ma13163566

**Published:** 2020-08-12

**Authors:** Yawen Wang, Zahra Gohari Bajestani, Jérôme Lhoste, Sandy Auguste, Annie Hémon-Ribaud, Monique Body, Christophe Legein, Vincent Maisonneuve, Amandine Guiet, Sylvette Brunet

**Affiliations:** 1 Institut de Chimie et Matériaux de Poitiers UMR 7285, University of Poitiers, CEDEX 9, 86073 Poitiers, France; yawen.wang@univ-poitiers.fr; 2 Institut des Molécules et Matériaux du Mans (IMMM), UMR 6283 CNRS, Le Mans Université, CEDEX 9, 72085 Le Mans, France; zahra.goharibajestani@univ-lemans.fr (Z.G.B.); jerome.lhoste@univ-lemans.fr (J.L.); sandy.auguste@univ-lemans.fr (S.A.); annie.ribaud@univ-lemans.fr (A.H.-R.); monique.body@univ-lemans.fr (M.B.); christophe.legein@univ-lemans.fr (C.L.); vincent.maisonneuve@univ-lemans.fr (V.M.)

**Keywords:** inorganic fluorides, nanoparticles, nanoparticle size, microwave synthesis, solvothermal route, catalytic performance, BET surface area, XRPD data, TEM data, solid-state NMR

## Abstract

High-specific-surface-area MgF_2_ was prepared by microwave-assisted solvothermal synthesis. The influences of the solvent and the magnesium precursors, and the calcination atmospheres, on the nanoparticle sizes and specific surface areas, estimated by X-Ray Powder Diffraction, N_2_ sorption and TEM analyses, were investigated. Nanocrystallized (~7 nm) magnesium partially hydroxylated fluorides (MgF_2−x_(OH)_x_) with significant specific surface areas between 290 and 330 m^2^∙g^−1^ were obtained. After activation under gaseous HF, MgF_2−x_(OH)_x_ catalysts underwent a large decrease of both their surface area and their hydroxide, rates as shown by their ^19^F and ^1^H solid-state NMR spectra. Expect for MgF_2_ prepared from the acetate precursor, an activity of 30–32 mmol/h∙g was obtained which was about 40% higher compared with that of MgF_2_ prepared using Trifluoroacetate method (21.6 mmol/h∙g).

## 1. Introduction

In the last few decades, numerous works have been devoted to the synthesis and characterization of metal nanofluorides due to their prospective applications in photonics, biosensing and the development of the new lubricants and high temperature superconductor devices and catalysis [1]. In particular, nanoscopic magnesium fluoride MgF_2_ is frequently applied in antireflective coatings [2,3,4], functional ceramics [5] and catalysis [6,7,8,9]. Many different synthesis approaches have been developed for the preparation of MgF_2_ nanosized particles, such as mechanochemical synthesis [10], sol–gel processes (TFA [11,12] and fluorolytic [13,14,15,16,17,18]), the precipitation method [19,20,21,22,23], reversed micelle synthesis [24,25] and hydro(solvo)thermal synthesis [26,27,28,29,30]. In this last approach, the use of non-metal alkoxides precursors together with the microwave-assisted solvothermal method combines the advantages of rapid heating and the pressurized solvothermal process. This association leads to a high reaction rate and short reaction time for higher efficiency and energy savings. Furthermore, in comparison with conventional heating in a classical furnace, microwave irradiation allows one to rapidly reach a homogeneous temperature, limiting the crystalline growth and consequently increasing of the specific surface areas of the synthesized materials [31]. For example, MgF_2_ nanoparticles were prepared using a mixture of magnesium acetate, HF and isopropanol treated at 90 °C for 30 min with a specific surface area of 116 m^2^∙g^−1^ [26,27]. This high surface area can be explained by the microwave/precursors–solvent interaction governed by two main processes involving in the heating mechanism [31]. The first process results in the alignment of the dipoles or ions in the electric field inducing molecular friction: it is the case of solvents with a dipolar moment and HF solution. The second, ionic conduction, is due to the presence of ions (Mg^2+^ and F^−^). These charged particles oscillate back and forth, collide with neighboring molecules and thus create heat. However, until now, no systematic study on the impacts of the solvent and the magnesium precursor on crystal growth to obtain a large surface area and their influences on the OH rates of MgF_2−x_(OH)_x_ nanoparticles obtained via microwave-assisted solvothermal synthesis has been published. In this paper, we report such a study through XRPD, N_2_ sorption, TEM and ^19^F and ^1^H solid-state NMR investigations. Furthermore, the influences of the different treatment atmospheres on the structure and surface properties were explored, especially under HF gas since catalytic gas-phase fluorination with anhydrous HF is a crucial process with which to introduce C–F bonds into organic compounds in industry [32]. More particularly, the effect of the HF gas treatment was studied by solid-state NMR and by CO adsorption followed by IR spectroscopy to quantify the number and strength of the Lewis active sites. Finally, the catalytic performances of these various MgF_2_ nanoparticles for the gas-phase fluorination of 2-chloropyridine as the model molecule are also reported.

## 2. Experimental Part

### 2.1. MgF_2_ Nanoparticles—Catalyst Preparation

Magnesium acetate (Mg(C_2_H_3_O_2_)_2_·4H_2_O, Alfa Aesar, Schiltigheim, France), magnesium nitrate (Mg(NO_3_)_2_·6H_2_O, Alfa Aesar), magnesium carbonate hydroxide (Mg_5_(CO_3_)_4_(OH)_2_.4H_2_O, Acros Organics, Noisy-le-Grand Seine-Saint-Denis, France) and magnesium chloride (MgCl_2_·6H_2_O, Alfa Aesar), were employed as the starting magnesium precursors and used without any prior purification. Solvents such as *^i^*PrOH, EtOH, MeOH, EG and DMSO were used as purchased by Sigma-Aldrich (Saint Quentin Fallavier, France). Hydrofluoric acid solution (15 mmol, 27.6 mol·L^−1^ (HF 40%) was provided by Riedel De Haen (Paris, France).

Magnesium fluoride nanoparticles were prepared by microwaves-assisted solvothermal route using different solvents (distilled water, *^i^*PrOH, EtOH, MeOH, EG and DMSO) and different magnesium precursors: carbonate (Mg(C_2_H_3_O_2_)_2_·4H_2_O), nitrate (Mg(NO_3_)_2_·6H_2_O), carbonate (Mg_5_(CO_3_)_4_(OH)_2_·4H_2_O) and chloride (MgCl_2_·6H_2_O) (Figure S1).

In a typical synthesis, 0.01 mol of magnesium precursor was introduced in a Teflon^®^ chamber (CEM Corp., Saclay, France); reactor 2 mL of the desired solvent was added under stirring. After few mins of stirring, 0.88 mL of aqueous hydrofluoric acid “HF 40%” (15 mmol, 27.6 mol·L^−1^) with a [F]/[Mg] molar ratio equal to 2, and 8 mL of solvent, were alternately added. The reactor was placed on the microwave’s carousel and connected to the temperature and pressure sensors of a MARS-5 microwave digestion system (CEM Corp., Saclay, France). The temperature was raised to 90 °C with a heating time of 5 min and was maintained for 30 min. After cooling to room temperature, the resulting product in gel form was collected and washed three times with EtOH by centrifugation, and the washed gel was dried in an oven at 80 °C. White powder of MgF_2_ nanoparticles was obtained. MgF_2_ prepared with different precursors is labelled MgF_2_-acetate, MgF_2_-carbonate, MgF_2_-chloride or MgF_2_-nitrate respectively. The effect of solvent was determined using magnesium acetate as precursor, and the effect of the magnesium precursor (acetate, carbonate, nitrate and chloride) using methanol as the solvent. The MgF_2_ samples prepared from various precursors in methanol were thermally treated under air flow and pure O_2_ at 350 °C for 2 h.

### 2.2. HF Activation and Catalytic Measurements

As described previously [12,32], the activation by HF and the catalytic activity performances of the various MgF_2_ materials for the transformation of 2-chloropyridine (2ClPy) were measured in a fixed-bed reactor. The different MgF_2_ samples were diluted with 6 cm^3^ of Lonza graphite (size grains between 125 and 200 µm). Firstly, the catalyst was activated in situ by HF under nitrogen (N_2_/HF molar ratio: 1/4) for 1 h at 350 °C (activation step). Then, 2-chloropyridine was introduced into the reactor using a syringe pump. The partial pressures of the various components were 0.806 bar for HF, 0.075 bar for 2-chloropyridine and 0.132 bar for nitrogen (HF/2ClPy/N_2_: 10.8/1/1.7). The organic gas products were trapped into 1,2-dichloroethane. HF and HCl were quenched in water at the outlet of the reactor. The organic components were analyzed with a Scion 456 gas-phase chromatograph (Bruker, Billerica, MA, USA) equipped with a DB5 capillary column (inside diameter: 0.2 mm; thickness film: 1 µm; length: 30 m). The oven temperature was raised from 100 to 200 °C at a rate of 5 °C·min^−1^.

The catalysts’ performances were compared at iso-conversion of 2-chloropyridine lower than 25% in order to be in a differential regime. In all cases, only 2-fluoropyridine (2FPy) was observed as a reaction product and HCl as by-product. Thus, the selectivity towards 2-fluoropyridine was equal to 100% and the conversion of 2-chloropyridine corresponded to the 2-fluoropyridine yield. In these experiments, the molar balance was always higher than 90%. No thermal decomposition of 2-chloropyridine was observed.

The catalytic activity A (mmol·h^−1^·g^−1^) was defined as the conversion of 2-chloropyridine multiplied by the flow of 2-chloropyridine (mol·h^−1^) and divided by the mass of catalyst. The intrinsic catalytic activity Ai (mmol h^−1^∙m^−2^) was calculated by taking into account the specific surface area of the catalyst after the activation step by HF. The turn over frequency, TOF (h^−1^), was calculated from the catalytic activity, A (mmol h^−1^∙g^−1^), divided by the number of actives sites defined as coordinately unsaturated metallic sites Qs (µmol∙g^−1^) measured by CO adsorption followed by IR spectroscopy.

Pure O_2_ (O_2_ ≥ 99.5%, 200 bar, L50) and gaseous HF (cylinder of 800 g) were provided by Air Liquid (Paris, France) and the 2-chloropyridine by Sigma-Aldrich.

### 2.3. Characterizations

All MgF_2_ samples (solvent, precursors and calcination effect) were characterized by X-Ray Powder Diffraction (XRPD) (PANalytical, Limeil Brévannes, France), N_2_ sorption, scanning electronic microscopy (SEM) (JSM 6510 LV, JEOL, Croissy sur Seine, France) and transmission electron microscopy (TEM) (JEOL JEM 2100 HR, JEOL, Croissy sur Seine, France) before and after the activation step at 350 °C under HF to follow the crystallinity and the morphology evolution of the MgF_2_ nanoparticles. The numbers of active sites and the strengths of their Lewis acidity were titrated by CO adsorption followed by infrared.

X-ray powder diffraction (XRPD) patterns of the MgF_2_ nanoparticles were recorded with a PANalytical θ/θ Bragg–Brentano Empyrean diffractometer (CuKα_1+2_ radiations) equipped with the PIXcel1D detector. Data were collected in the [15–110°] 2θ scattering angle range for a total acquisition time of 3 h with a 0.499° step. The NIST standard reference material LaB_6_ (NIST SRM 660b) was used to take account the instrumental broadening. The XRPD pattern of LaB_6_ was recorded with the same data collection conditions (step and counting time) used for the analysis of MgF_2_ samples. XRPD patterns were refined by using Le Bail method [33] implemented in the Fullprof program [34]. The X-ray line broadening due to the nanometric size of the sample contribution was calculated by using Thompson–Cox–Hastings pseudo-Voigt function [35] that includes size and strain-broadening terms for both Lorentzian and gaussian components. The two parameters (Y and F) of the Lorentzian component of this function were refined to calculate the apparent crystallite size <L> with Fullprof using Langford’s method [36]. The diameter DXRD of the spherical particles is related to <L> by the following formula DXRD = 4/3×<L>.

Scanning electronic microscopy (SEM) images of the powders were obtained using a JEOL microscope (JSM 6510 LV, Croissy sur Seine, France). Acceleration voltages varied between 20 and 30 kV as a function of the analyzed samples. Elementary quantitative microanalyses were performed using an Energy dispersive X-ray (EDX) OXFORD detector (AZtec software) (OXFORD Instruments, Gometz La Ville, France).

Transmission electron microscopy (TEM) study was performed on a JEOL JEM 2100 HR electron microscope (JEOL, Croissy sur Seine, France) operating at 200 kV. The samples for transmission electron microscopy investigation were prepared by ultrasonically dispersing each raw powder in ethanol, depositing a drop of the resulting suspension onto a holey carbon-coated copper grid and finally drying the grid in air. Mean diameters of the nanoparticles (D_TEM_) were deduced by statistical evaluation of about 100 particles using ImageJ software [37]. For all measurements, the standard deviation was around 25%.

The specific surface areas were measured at 77 K using a TriStar II 3020 (Micrometrics, Merignac, France). The MgF_2_ samples were degassed under vacuum at 100 °C for 12 h prior to measurement. The specific surface areas were calculated using the Brunauer–Emmett–Teller (BET) method. For accurate determination of specific surface areas as low as 30 m^2^·g^−1^, a minimum of ≈ 350 mg of powder had to be used, as the lowest surface measured using a TriStar II 3020 is around 10 m^2^ in the cell.

After synthesis and calcination under air and O_2_ of MgF_2_ powders, Fourier transform infrared spectroscopy (FT–IR) spectra were collected in air at room temperature with a Bruker ALPHA FT–IR spectrometer (Bruker, Wissembourg, France) equipped with the Platinum QuickSnap ATR sampling module. The spectral resolution was 4 cm^−1^ in the 400–4000 cm^−1^ range. Twenty-five consecutive scans were averaged to obtain a single spectrum. A reference IR spectrum was collected in the same conditions with an empty cell and subtracted from specimen spectra to remove H_2_O(g) and CO_2_(g) contributions.

The CO adsorption was carried out using a ThermoNicolet NEXUS 5700 (ThermoFisher Scientific, Paris, France) spectrometer with a resolution of 2 cm^−1^ and 64 scans per spectrum were collected. Samples were pressed into thin pellets (10–60 mg) with diameter of 16 mm and activated in situ during one night at 350 °C under high vacuum (≈ 10^−6^ bar). After cooling down the samples to room temperature, the cell was cooled down with liquid nitrogen to 100 K. A background spectrum was collected which was then subtracted from the other spectra obtained after CO adsorption. Then, successive doses of CO were introduced quantitatively, and an infrared spectrum was recorded after each adsorption until saturation. The final spectrum was recorded with 1 Torr of CO at equilibrium pressure (saturation). All spectra were normalized to an equivalent sample mass (25 mg). The quantification of the amount of CO adsorption corresponding to the titration of active Lewis acid sites Qs (µmol∙g^−1^) was carried out by the integration of the total area of the IR bands at saturation between 2100 and 2200 cm^−1^ using the molar absorption coefficient ε of MgF_2_ [38].

Thermogravimetric analyses were either performed with a TGA-TA Instruments SDT Q600 (TA, Paris, France) between 25 and 900 °C under synthetic air flow with a heating rate of 5 °C min^−1^ or on a Netzch STA 449 F3 (Netzch, Dardilly, France) coupled with a QMS 403 C mass spectrometer (1–200 amu mass range) under a N_2_/air atmosphere with a heating rate of 10 °C/min. A continuous analysis of HF, F^−^, H_2_O and HO^−^ rates was carried out for MgF_2_-acetate directly after synthesis.

The elemental analysis of carbon of the metal fluorides was carried out with an elementary analyzer (NA2100 analyzer, CE instruments, Paris, France).

^1^H and ^19^F solid-state MAS NMR experiments were performed on a Bruker Avance III spectrometer operating at 7.0 T (^1^H and ^19^F Larmor frequencies of 300.1 and 282.4 MHz, respectively), using a 1.3 mm CP-MAS probe head. The ^1^H and ^19^F MAS spectra were recorded using a Hahn echo sequence with an interpulse delay equal to one rotor period. The 90° pulse lengths were set to 2.4 and 1.25 μs; the recycle delays were set to 20 s and 900 s; and 8 to 192 and 32 (or 48) transients were accumulated for ^1^H and ^19^F, respectively. ^1^H and ^19^F spectra refer to TMS and CFCl_3_, respectively, and they were fitted by using the DMFit software (dmfit#20200306, CEMHTI - CNRS - UPR3079, Orléans, France) [39].

## 3. Results and Discussion

### 3.1. Synthesis of MgF_2_ Nanofluorides

MgF_2_ nanofluorides were synthesized via the facile and rapid microwave-assisted solvothermal method using aqueous HF (HF 40%) as the fluorinated agent. The impacts of the solvent, the magnesium precursors, the calcination atmospheres (air and O_2_) and the activation step by HF, on the composition, the particle size and the specific surface area are reported. Assuming a stoichiometric reaction, the reaction can be described by Equation (1) for the acetate, chloride and nitrate precursors and by Equation (2) for the carbonate magnesium precursor.

(1) MgX_2_ + 2 HF → nano-MgF_2_ + 2 HX,

(2) Mg_5_(CO_3_)_4_(OH)_2_ + 10 HF → nano-MgF_2_ + 4 CO_2_ + 6 H_2_O,

The impacts of the solvent dielectric properties were studied using magnesium acetate (Mg(C_2_H_3_O_2_)_2_) as the precursor. Indeed, it has been shown that the nature of the solvent significantly influences the size of the nanoparticle prepared by solvothermal synthesis [40]. MeOH, H_2_O, *^i^*PrOH, EtOH, EG and DMSO were selected and their respective dielectric constants, dielectric losses and loss angles tan δ (tan δ = ε″/ε′ with ε′, the dielectric constant and ε″, the dielectric loss) are presented in Table 1.

### 3.2. MgF_2_ Nanofluoride’s Characterization by XRPD, N_2_ Sorption, FT–IR and TEM

**Figure 1:**
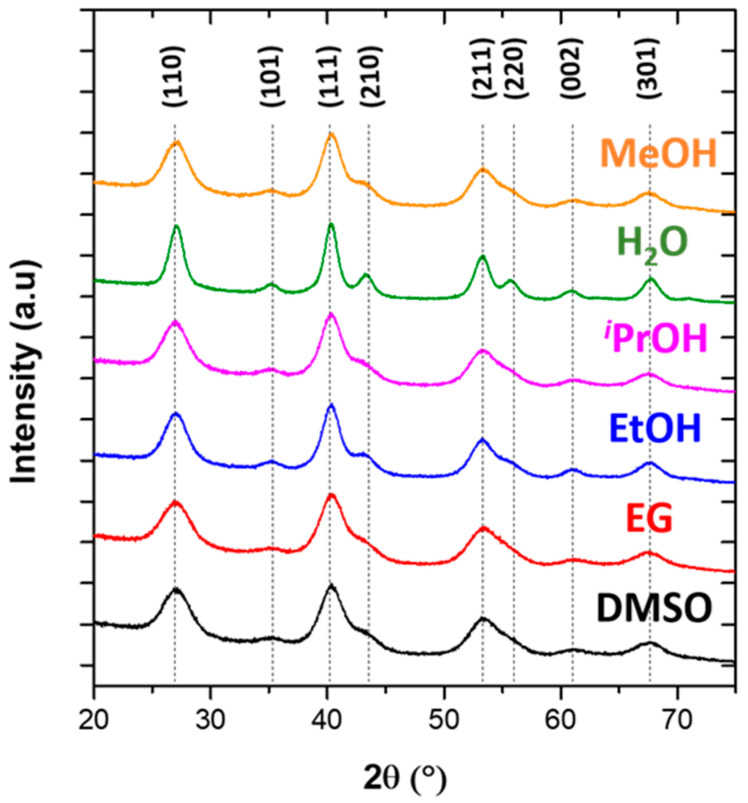
X-Ray Powder Diffraction (XRPD) patterns of MgF_2_ nanoparticles prepared by microwaves-assisted solvothermal synthesis using H_2_O, *^i^*PrOH, EtOH, EG or DMSO as solvent.

XRPD confirmed the rutile type crystal structure of MgF_2_ (space group P4_2_/mnm (n°136), 20513-ICSD) independently of the solvent used (Figure 1).

Among all samples, MgF_2_ nanoparticles prepared in MeOH, ^i^PrOH, EtOH and DMSO solvents presented the highest specific surface areas (S_BET_) of between 325 and 345 m^2^·g^−1^ (Table 2), related to the lower particle diameters between 4 and 6 nm, determined by Le Bail refinement from the XRPD pattern (D_XRD_). Those highest specific surface areas can be explained by the high tan δ values for those solvents (Table 1). The lowest specific surface area measured for the sample prepared in H_2_O corresponds to the formation of particles of larger size (8–11 nm). This phenomenon can be explained by the lower tan δ value of H_2_O. In the case of MgF_2_-EG, the specific surface area is surprisingly low despite the small nanoparticle size (6–7 nm) and the highest tan δ (tan δ = 1.349). This result could be related to the presence of EG residue on the nanoparticle’s surface evidenced by the FT–IR analysis; the EG absorption bands [42] at 1050 cm^−1^ (ν(C–O) stretching)) and at 950 cm^−1^ (ν(C–C) stretching) could be observed (Figure S2). EG might be embedded in the NPs and have a negative effect on the NP’s surface. To conclude, as the reaction yields for DMSO and EtOH were very low (less than 50%), MeOH was selected as an optimized solvent for the synthesis of MgF_2_ nanoparticles with high specific surface areas and the smallest particle size, as demonstrated in the case of nanostructured β-AlF_3−x_(OH)_x_ [43]. For all spectra, the two bands observed at 3393 cm^−1^ and 1598 cm^−1^ correspond to hydroxyl groups of H_2_O and EtOH molecules adsorbed on the surfaces of the nanoparticles.

Regarding the influences of the magnesium precursors on the MgF_2_ nanoparticle features, four magnesium precursors (magnesium acetate, carbonate, chloride and nitrate) were selected. Transparent MgF_2_ gels were obtained except for MgF_2_-carbonate, for which an opaque white gel was observed (Figure S3). The MgF2 nanoparticles’ purity was first controlled by XRPD analysis, and XRPD profile refinements confirm the rutile type crystal structure of MgF2 (space group P42/mnm (n°136), 20513-ICSD) independently of the precursor used (Figure S4).

The purity was also confirmed by the FT–IR analyses, as illustrated in Figure S5. All spectra show a strong adsorption band at 460 cm^−1^ corresponding to the stretching vibration ν of the Mg-F bond and two bands centered at 1600 and 3700 cm^−1^ assigned to the bending vibration *δ* and the stretching vibration ν of water molecules [29]. In the case of MgF_2_-nitrate, two extra bands emerged at 1400 cm^−1^ and 1350 cm^−1^ corresponding to nitrate residue (ν stretching) at the particle surface. The low carbon amount analyzed by elemental analysis (<3%) confirmed also the bright white color of the MgF_2_ nanoparticle powders (Figure S6). High specific surface areas (S_BET_), ranging from 237 m^2^∙g^−1^ to 372 m^2^∙g^−1^, were obtained for the four MgF_2_ samples (Table 3).

**Figure 2:**
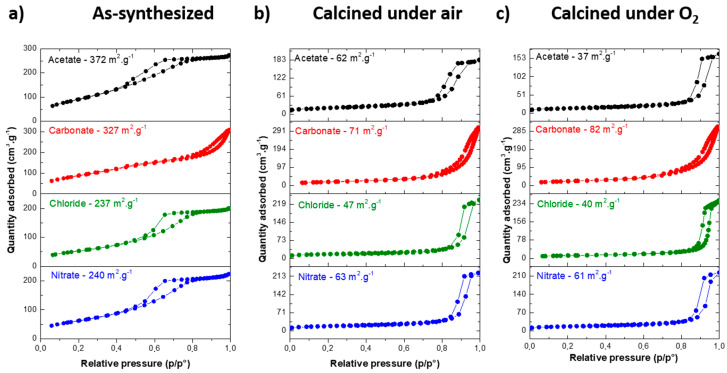
N_2_ adsorption and desorption isotherms of (**a**) as-synthesized; (**b**) calcined under air (350 °C, 2 h) and (**c**) calcined under O_2_ (350 °C, 2 h) MgF_2_ prepared from acetate, carbonate, chloride and nitrate magnesium precursors.

Before any thermal treatment, all isotherms belonged to type IV according to IUPAC classification, i.e., the type characteristic of mesoporous materials (Figure 2a). The isotherms measured for MgF_2_ nanoparticles prepared from acetate, chloride and nitrate magnesium precursors are similar to the hysteresis loop of type H_2_ [44,45]. Such pore structures can be regarded as intergranular porosity. Surprisingly, a different shape for the hysteresis loop was observed for MgF_2_-carbonate. This shape tends to be similar to the H3 type observed for aggregates.

**Figure 3:**
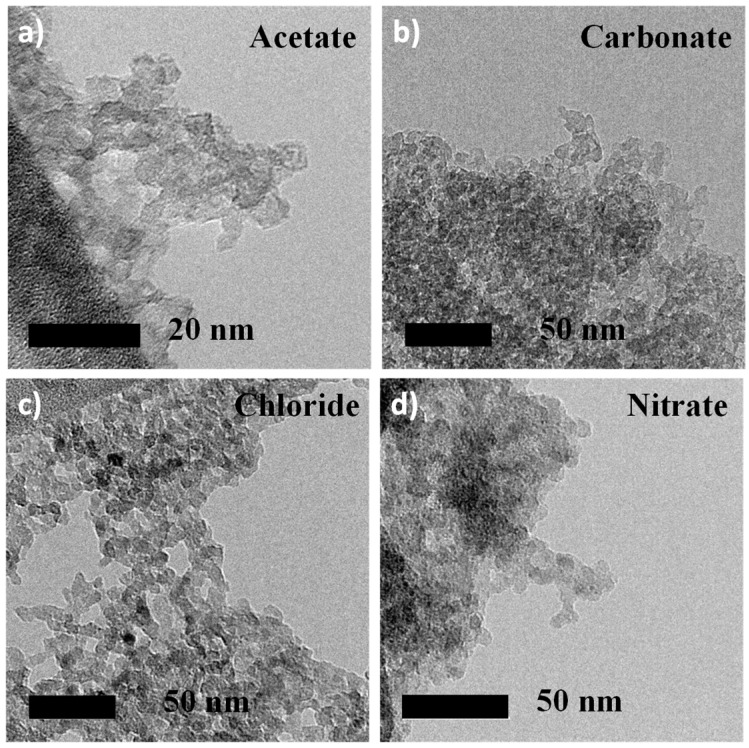
TEM of MgF_2_ nanoparticles prepared from acetate (**a**), carbonate (**b**), chloride (**c**) and nitrate (**d**) magnesium precursors.

The size, morphology and structure of the particles were characterized by TEM (Figure 3), thereby revealing that the MgF_2_ nanoparticles were irregularly shaped with average crystallite sizes of 5 to 10 nm, consistent with those obtained from XRPD Le Bail refinements (Table 4, results of a refinement are given as an example in Table S1).

Selected area electron diffraction (SAED, Figure S7) performed on TEM confirmed also the crystallinity of the MgF_2_ nanoparticles, as all rings of the SAED patterns were well indexed to the crystal planes (110), (011), (111) and (121) of the rutile type MgF_2_ structure. No lattice fringe could be observed at high resolution, as MgF_2_ nanoparticles were not stable at high magnification under the electron beam as it induced the departure of the water molecules. Among these four pure MgF_2_ samples, MgF_2_ nanoparticles prepared from magnesium acetate precursor exhibited the highest specific surface areas (S_BET_) (372 m^2^∙g^−1^, Table 2) related to the lower particle diameters of between 4 and 5 nm, determined by Le Bail refinement form the XRPD pattern (D_XRD_).

To probe the thermal stability of the particles synthesized from different precursors, thermal treatments were performed under air and under O_2_, at 350 °C for 2 h. The temperature of 350 °C was chosen, as it is the temperature of the fluorination reaction of 2-chloropyridine. A drop of specific surface area for all samples was observed after the calcination in air and in O_2_ (Table 3), and a significant change in the hysteresis loop was induced (Figure 2b,c). Indeed, after calcination in air, the specific surface areas were around 60–70 m^2^∙g^−1^ except for the sample prepared using magnesium chloride as a precursor (47 m^2^∙g^−1^). Differences were more pronounced after the calcination under O_2_, for which the lower specific surface areas were observed for the samples prepared using acetate and chloride magnesium precursors and the highest for those prepared using nitrate and carbonate magnesium precursors. Except for MgF_2_-carbonate (D ≈ 9 nm), this decrease corresponds to a nanoparticle growth induced by the thermal calcination in air and in O_2_ (D_XRD_ and D_TEM_ ≈ 15–20 nm, Table 4). Despite the stability of the rutile structure, a decrease of the specific surface area was also observed after the activation by HF under operating conditions (350 °C) of the catalytic fluorination of 2-chloropyridine.

**Figure 4:**
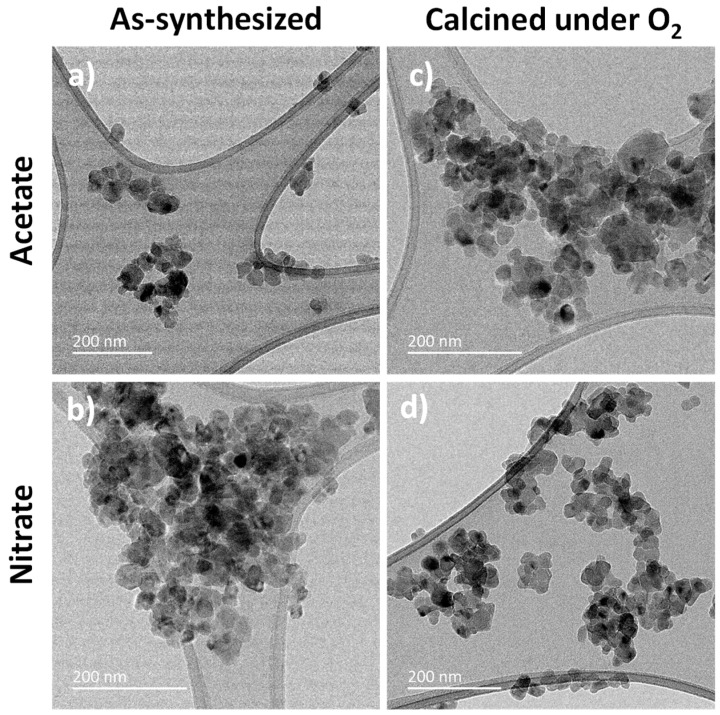
TEM of as-synthesized (**a**,**b**) and calcined in O_2_ (**c**,**d**) MgF_2_ prepared from acetate and nitrate magnesium precursors, after the HF activation step.

**Figure 5:**
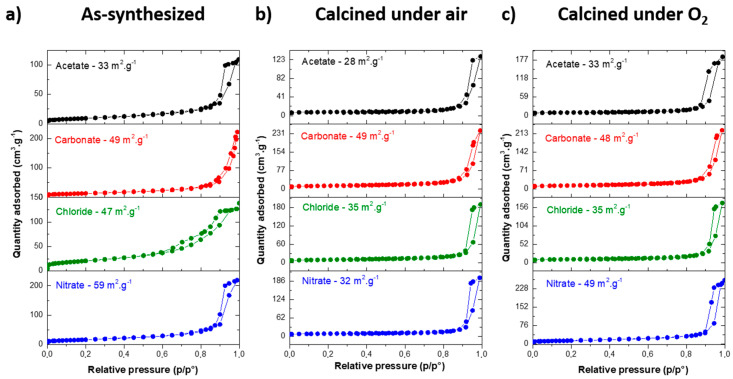
N_2_ adsorption and desorption isotherms of (**a**) as-synthesized; (**b**) calcined in air and (**c**) calcined in O_2_ MgF_2_ prepared from acetate, carbonate, chloride and nitrate magnesium precursors, after HF activation.

In fact, whatever the magnesium precursors used, similar specific surface areas were observed after the activation step by HF of the as-synthesized samples and after the calcination step under air and under O_2_. This demonstrates that the calcination step did not stabilize the MgF_2_ samples and is not crucial before the activation step by HF gas. Except for the sample prepared with magnesium acetate as the precursor (33 m^2^∙g^−1^), the microwave-assisted solvothermal method using MeOH as solvent allowed us to obtain higher-specific-surface-area MgF_2_ catalysts (50–60 m^2^∙g^−1^) after the activation step by HF (Figure 4, Figure 5 and Figure S8) in comparison with MgF_2_ prepared from commercial magnesium oxide (16 m^2^∙g^−1^) or carbon-free MgF_2_ prepared by using TFA method (33 m^2^∙g^−1^) [12].

### 3.3. MgF_2_ Nanofluoride’s Characterization by ^19^F and ^1^H Solid-State MAS NMR

**Figure 6:**
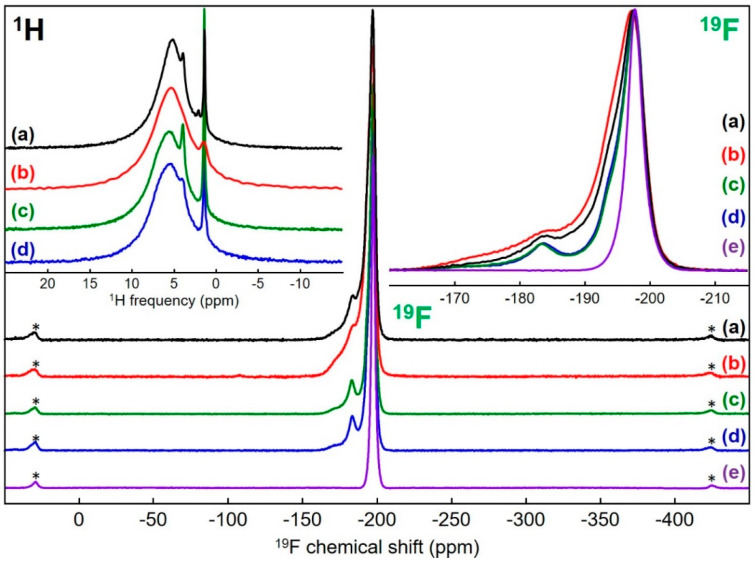
^19^F and ^1^H (in the inset on the left) solid-state MAS (64 kHz and 60 kHz, respectively) NMR spectra of MgF_2_ samples prepared from (**a**) acetate, (**b**) carbonate, (**c**) chloride and (**d**) nitrate magnesium precursors, before the HF activation step and for ^19^F of (**e**) a micro crystalline sample of MgF_2_. The isotropic lines of the ^19^F spectra are expanded in the inset on the right. The star symbols on the ^19^F spectra indicate the spinning sidebands.

The structural investigations were completed by ^19^F and ^1^H solid-state MAS NMR studies before and after the activation step by HF for MgF_2_ samples prepared from acetate, carbonate, chloride and nitrate precursors. For the rutile type crystal structure of MgF_2_ involving a single F crystallographic site [46], a single ^19^F NMR line is observed on its spectrum [47]. The ^19^F spectra of the samples before activation show a main line with a maximum at a similar isotropic chemical shift value (*δ*_iso_~−197.7 ppm) but also a shoulder towards higher *δ*_iso_ values, and even, distinctly, a second contribution at around −185 ppm (Figure 6, fits and results of these fits given in Figure S9 and Table S2).

**Figure 7:**
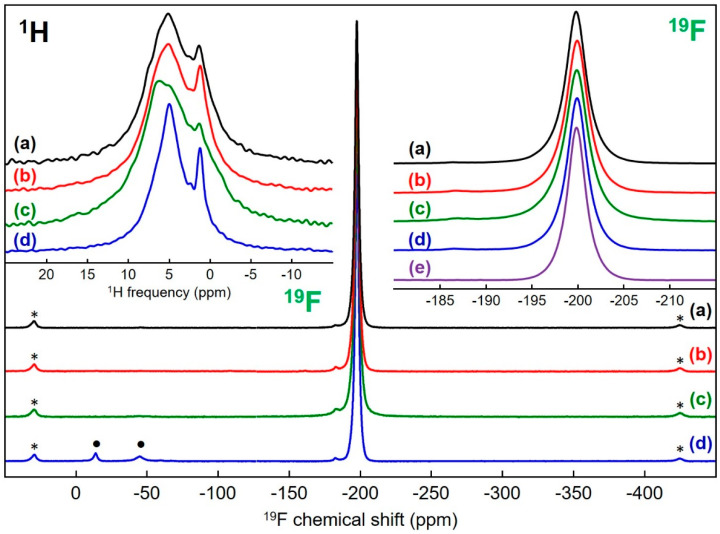
^19^F and ^1^H (in the inset on the left) solid-state MAS (64 kHz and 60 kHz, respectively) NMR spectra of MgF_2_ samples prepared from (**a**) acetate, (**b**) carbonate, (**c**) chloride and (**d**) nitrate magnesium precursors, after the HF activation step. The isotropic lines of the ^19^F spectra are expanded in the inset on the right and compared with that of (**e**) a micro crystalline sample of MgF_2_ recorded under the same conditions. The star symbols on the ^19^F spectra indicate the spinning sidebands. The dot symbols on the ^19^F spectra of (**d**) indicate fluoride impurities.

The spectra of the samples after activation show far less pronounced shoulders (Figure S10) but remain asymmetrical (three contributions with close *δ_i_*_so_ are necessary to reconstruct them perfectly, (Figure 7; fits and results of these fits are given in Figure S11, Figure S12 and Table S3)). Moreover, compared with the ^19^F NMR line of a MgF_2_ microcrystalline sample [48], these lines are broader (line widths of 876, 930, 1000, 853 and 795 Hz for samples prepared from acetate, carbonate, chloride and nitrate precursors and micro crystalline, respectively). The broadening mirrors chemical shift distributions related to disorder around fluorine atoms due to the high specific surface area and partial hydroxylation. The shoulders or contributions at higher ^19^F *δ*_iso_ values are due to partial hydroxylation. The increase of the ^19^F *δ*_iso_ value with the hydroxide content, i.e., the number of hydroxide groups in the environment of the fluoride ion in hydroxyfluorides, is a well-known phenomenon which has been observed in magnesium hydroxyfluorides (up to 40 ppm) [49]. Nevertheless, due to the very large number of different environments of the fluoride ions in magnesium partially hydroxylated fluorides, MgF_2−x_(OH)_x_, the assignment of the additional lines seems to be unrealistic. Indeed, the environment of the site of the fluoride ion in MgF_2_ consists of three magnesium ions and eleven fluoride ions at four different distances (between 2.58 and 3.35 Å, Table S4) [46].

Then, depending on the positions occupied by the anions, there are several inequivalent FMg_3_F_11−y_(OH)_y_^6−^ (0 ≤ y ≤ 11) environments for each of the twelve y values. In any case, the proportion of fluoride ions in environments free of hydroxide ions (FMg_3_F_11_) can be roughly estimated within the range 35% to 50% for the samples before activation (Table S2) and is superior to 80% for the samples after activation (Table S3). The rates of –OH groups can be estimated by assuming a random distribution of OH^−^ and F^−^ ions at anionic sites (Table S5). Only rates ranging from 6% to 9% (0.12 ≤ x ≤ 0.18) for the samples before activation and below 2% (x < 0.04) for the samples after activation satisfy such proportions of FMg3F11 environment. The rates of −OH groups were therefore low in the as-prepared samples and very low after HF treatment, in agreement with Wuttke et al. regarding MgF_2−x_(OH)_x_ samples prepared by fluorolytic sol–gel synthesis [16]. In detail, for samples prepared from chloride and nitrate precursors which show similar spectra and the narrowest shoulders, similar OH rates around 6% are expected, substantially lower than those of the samples prepared from acetate and carbonate precursors, for which OH rates of 8% and 9% were respectively estimated. For the latter two samples, it should be noted that the formation of bonds between magnesium atoms on the surfaces of the nanoparticles and residual acetate and carbonate ions acting as ligands is likely and that the effect of such ligands on the ^19^F *δ_i_*_so_ values is expected to be similar to that of −OH groups; −OH group rates are potentially the sum of rates of −OH and −OCOCH_3_ or −OCO_2_ groups. Finally, for the as-prepared MgF_2−x_(OH)_x_ samples, it appears that specific surface areas and −OH group rates are not independent. Even if the sample with the highest –OH group rate did not have the largest surface area, a trend was observed with the two samples with the smallest (largest) surface areas and the smallest (largest) OH rates. After activation, whatever the precursor used, i.e., whatever the OH rate of the MgF_2−x_(OH)_x_ samples, similar OH rates below 2% are likely. Surprisingly, at first glance, the widths of the main ^19^F NMR lines and the areas are not classified in the same order. The narrowest main line was even observed for the sample prepared from nitrate precursor, which had the largest specific surface area, but this is consistent with the greatest relative intensity of the lines attributed exclusively to FMg_3_F_11_ environments.

The ^1^H solid-state MAS NMR spectra of the four as-prepared samples (Figure 6; fits and results of these fits in Figure S13 and Table S6) and after HF activation (Figure 7; fits and results of these fits in Figure S14 and Table S7) present one main broad contribution with *δ*_iso_ values maximally ranging from 5.0 to 5.8 ppm, assigned to absorbed H_2_O molecules and a smaller contribution with *δ*_iso_ values maximally ranging from 1.0 to 1.4 ppm, assigned to Mg_3_–OH environments [50]. However, the spectra of the samples before and after treatment are clearly differentiated by several characteristics, the most significant being the larger relative intensity of the contribution assigned to Mg_3_–OH environments after activation, largely offset by the large decrease of the intensity of the NMR spectra. This decrease is explained by a decrease of the amounts of adsorbed H_2_O molecules, related to the decrease of the surfaces of the nanoparticles, leading, despite a large number of scans, to rather low signal to noise ratios and confirming that, after activation, the amounts of residual –OH groups are small. The ^1^H NMR contributions are broadened by distributions in the chemical shifts reflecting heterogeneity of the local hydrogen environment. As for ^19^F, after activation, it was for the sample prepared from the nitrate precursor, that the broadening was the least, despite its higher specific surface area. Lastly, the ^1^H spectrum of the sample prepared from acetate precursor shows a line at 2.1 ppm (Table S6) which can be assigned to protons from residual −OCOCH_3_ group, probably bonded to magnesium atoms on the surfaces of the nanoparticles.

### 3.4. Properties of the Active Sites Characterized by Adsorption of CO as a Probe Molecule Followed by IR Spectroscopy

Finally, after the activation step by HF which led to the effective catalyst, the properties of the active sites (quantities and strength of Lewis acidity) involved in Cl/F exchanges were characterized by adsorption of CO as a probe molecule followed by IR spectroscopy. CO is a suitable probe molecule for the characterization of Lewis acidity because the (CO) frequency is very sensitive to the local cationic environment. After CO adsorption, this band shifts more or less depending on the strength of Lewis acidity: the stronger the Lewis acidity, the higher the shift. On the different studied catalysts, CO was adsorbed on under-coordinated metal sites of metal fluorides which were the Lewis acid sites. The change of the strength of Lewis acidity between each catalyst was practically studied. It is also important to note that CO can also be adsorbed on Bronsted acid sites. In the present work, no Bronsted acid sites were detected.

**Figure 8:**
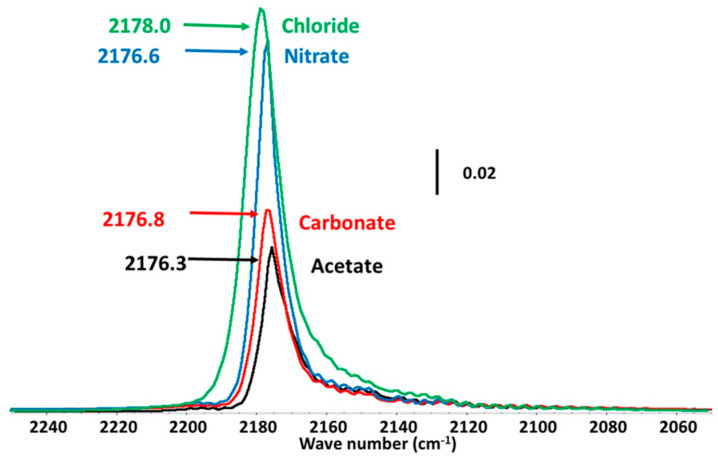
CO adsorption followed by FT–IR over as-synthesized MgF_2_ prepared from acetate, carbonate, chloride and nitrate magnesium precursors after the HF activation step.

As reported in Figure 8, no difference of wave numbers (2170 cm^−1^) was noticed for the samples corresponding to similar low Lewis acidity strengths for all unsaturated metallic sites. Regarding the quantification (Table 5), it is difficult to establish a trend given the uncertainties. The number of sites appears to be constant except for three samples for which the values are higher. These experiments were reproduced and the results confirmed. The adsorption of several CO molecules on the same site may be suggested as reported by Wuttke et al. [51]. The formation of complexes between CO and metallic centers may be also considered. On the other hand, the concentration of sites C_sites_ was constant (1.8–2.0 µmol∙m^−2^) whatever the precursor used except after the treatment in air.

### 3.5. Catalytic Performances

The performances of the various MgF_2_ samples were measured for the transformation of 2-chloropyridine at 350 °C under atmospheric pressure (Table 6). All MgF_2_ catalysts are active for the fluorination of 2-chloropyridine with a selectivity towards 2-fluoropyridine of 100%. Expect for MgF_2_ prepared from the acetate precursor, an activity of 30–32 mmol∙h^−1^∙g^−1^ was obtained, which was about 40% higher than that of MgF_2_ prepared using TFA method (21.6 mmol·h^−1^∙g^−1^) [12]. This gain of activity was due to the increase of the specific surface area (from 33 m^2^∙g^−1^ to 48 m^2^∙g^−1^). Indeed, in both cases, the activity per square meter was around 0.7 mmol∙h^−1^∙g^−1^. The lower activity of the MgF_2_ prepared from the acetate precursor could be explained from the different thermal behaviors of the as-synthesized MgF_2_ nanoparticles (Figure S15). For all, a first weight loss of ≈20% related to the departure of residual solvent and HF and –OH group was observed.

In the case of MgF_2_-acetate, a supplementary weight loss was clearly detected at higher temperatures. In order to get more information, MS-TGA analysis was performed on this sample (Figure S16). It confirmed the release of H_2_O or –OH groups (m/z 18), F^–^ groups (m/z 19) and CH_3_^+^ groups between 110 °C and 150 °C. The CH_3_^+^ group came from acetic acid formed during the reaction between acetate precursor and HF. The second CH_3_^+^ peak observed at 440 °C was probably due to acetate groups strongly coordinated to the metal ions at the surface, as confirmed by ^19^F NMR. These remaining acetate groups could block the active sites lowering the catalyst activity. Regarding the activity per square meter, no difference of activity (0.7–0.8 mmol∙m^2^∙g^−1^) was noticed according to the similar specific areas, nanoparticle sizes and morphologies of the different MgF_2_ samples after HF treatment. Finally, the activity per site (TOF defined as A/Q_sites_ ratio) was in general the same, whatever the magnesium precursor, except for three samples for which the TOF was lower (around 200 h^−1^ instead 350 h^−1^). This corresponded also to an abnormally high CO adsorption.

## 4. Conclusions

In this work, various parameters involved in the synthesis of MgF_2_ by microwave-assisted solvothermal synthesis were investigated. Methanol as a solvent provided significant yields, small nanoparticle size and the highest specific surface area. Whatever the magnesium precursors, hydroxylated magnesium fluoride MgF_2−x_(OH)_x_ compounds with OH rates probably ranging from 6% to 9% (0.12 ≤ x ≤ 0.18) and with large specific surfaces ranging from 237 m^2^∙g^−1^ to 372 m^2^∙g^−1^ (from magnesium acetate precursor) were obtained. All were strongly impacted by the HF activation step, during which significant decreases of both the specific surface area, ranging from 28 m^2^∙g^−1^ to 59 m^2^∙g^−1^, and the OH rates (below 2%) were highlighted. Despite this drop of the specific surface area, microwave-assisted solvothermal synthesis using non-alkoxide precursors allows the rapid, straightforward and cheap preparation of scalable MgF_2_ catalysts for the fluorination of 2-chloropyridine with a higher activity (40%) compared with that of MgF_2_ prepared using the TFA method.

## Figures and Tables

**Table 1:** Dielectric constants (ε′), dielectric losses (ε″) and loss tangents (tan δ = ε″/ε′) of the various solvents (MeOH, H_2_O, ^*i*^PrOH, EtOH, EG and DMSO).

Solvent	ε′	ε″	tan δ
MeOH	34.0	21.5	0.632
H_2_O	80.4	9.9	0.123
*^i^*PrOH	18.3	14.6	0.798
EtOH	24.3	22.9	0.942
EG	37.0	49.9	1.349
DMSO	45.0	37.1	0.824

The loss angle tan δ is a measure of reactance (resistance in a capacitor) of a molecule. A material with δ = 0 is completely transparent to microwave irradiation and tan δ is infinite for a perfectly absorbing material. Solvents with tan δ around 1 are remarkable microwave absorbers [41].

**Table 2:** Impacts of the solvent on the specific surface areas (S_BET_) and particle diameters determined by XRPD (D_XRD_) of the nanoparticles of MgF_2_ and yield of the synthesis.

Solvent	S_BET_ (m^2^·g^−1^)	D_XRD_ (nm)	Yield (%)
MeOH	345	4	~100
H_2_O	237	11	~100
*^i^*PrOH	325	7	~95
EtOH	334	6	~45
EG	267	6	~30
DMSO	340	5	~20

**Table 3:** Impacts of the magnesium precursors (acetate, carbonate, chloride, nitrate) and of the treatments (air, O_2_) on the specific surface areas (S_BET_, m^2^∙g^−1^) of the MgF_2_ samples, before and after HF activation.

Sample		Acetate	Carbonate	Chloride	Nitrate
As-synthesized	Before HF	372	327	237	240
	After HF	33	49	47	59
Air	Before HF	62	71	47	63
	After HF	28	49	35	32
O_2_	Before HF	37	82	40	61
	After HF	33	48	35	49

**Table 4:** Impacts of the precursors and of the treatments in air and in O_2_ on the MgF_2_ nanoparticle diameters, determined by XRPD (D_XRD_, nm) and TEM (D_TEM_, nm), before and after HF activation.

Sample		Acetate		Carbonate		Chloride		Nitrate	
		D_XRD_	D_TEM_	D_XRD_	D_TEM_	D_XRD_	D_TEM_	D_XRD_	D_TEM_
As-synthesized	Before HF	4	5	4	5	8	5	7	5
	After HF	35	36	19	21	19	2	27	28
Air	Before HF	14	-	9	-	15	-	14	-
	After HF	33	-	15	-	28	-	27	-
O_2_	Before HF	20	21	9	-	16	-	14	16
	After HF	27	28	16	-	25	-	24	27

**Table 5:** CO adsorption followed by IR of MgF_2_ samples after HF activation. Impacts of the magnesium precursors and of the calcination atmosphere (air and O_2_) on the number of acid sites (Q_sites_: µmol∙g^−1^) and their concentration (C_sites_: µmol∙m^−2^). Estimated relative uncertainty: 5%.

Sample	Acetate		Carbonate		Chloride		Nitrate	
	Q_sites_	C_sites_	Q_sites_	C_sites_	Q_sites_	C_sites_	Q_sites_	C_sites_
As-synthesized	64	1.9	87	1.8	177	2.4	106	1.8
Air	87	3.1	156	3.2	79	2.3	90	2.8
O_2_	68	2.1	151	1.8	78	1.7	96	2.0

**Table 6:** Impacts of the precursors and of the treatment in air and in O_2_ of different MgF_2_ samples after HF activation on the transformation of 2-chloropyridine (T = 350 °C, HF/2chloropyridine/N2: 7/1/1.7, isoconversion around 30%). A: Activity for the transformation of 2-chloropyridine: (a) mmol.h^−1^∙g^−1^; (b) mmol.h^−1^∙m^−2^; TOF (turn over frequency, h^−1^): A/Q_sites_ with A (activity, mmol∙h^−1^∙g^−1^); and Q_sites_ (µmol∙g^−1^), the amount of CO adsorbed followed by FT–IR. Estimated relative uncertainty: 15%.

Sample	Acetate			Carbonate			Chloride			Nitrate		
	A		TOF	A		TOF	A		TOF	A		TOF
	(a)	(b)	-	(a)	(b)	-	(a)	(b)	-	(a)	(b)	-
As-synthesized	26	0.75	406	32	0.66	370	30	0.64	170	29	0.49	270
Air	29	1.02	330	32	0.67	210	30	0.87	380	31	0.63	340
O_2_	25	0.77	370	32	0.83	210	31	0.89	400	31	0.63	320

## Supplementary Materials

The following are available online at https://www.mdpi.com/1996-1944/13/16/3566/s1. Figure S1: Representation of microwaves assisted synthesis of MgF_2_ nanoparticles. Figure S2: FT–IR spectra of MgF_2_ powders synthetized using H_2_O, *^i^*PrOH, EtOH, EG and DMSO as solvent. Figure S3: Pictures of the as-synthesized MgF_2_ nanoparticles as gel after the washing procedure and before drying to obtain a white powder. Figure S4: Le Bail refinements of MgF_2_ nanoparticles prepared from (**a**) acetate, (**b**) carbonate, (**c**) chloride and (**d**) nitrate magnesium precursors. Vertical markers give the Bragg peak positions of the crystalline structure of MgF_2_ (space group P4_2_/*mnm* (n°136)). Figure S5: FT–IR spectra of MgF_2_ nanoparticles prepared from acetate, carbonate, chloride and nitrate magnesium precursors and using MeOH as solvent. Figure S6: Pictures of MgF_2_ nanoparticles powders prepared from different precursors. Figure S7: SAED patterns of MgF_2_ nanoparticles prepared from (**a**) acetate, (**b**) carbonate, (**c**) chloride and (**d**) nitrate precursors. Figure S8: Nanoparticle sizes of MgF_2_ after HF treatment measured from TEM images. Figure S9: ^19^F MAS (64 kHz) experimental (blue line) and fitted (dashed red line) NMR spectra of the MgF_2_ samples prepared from (**a**) acetate, (**b**) carbonate, (**c**) chloride and (**d**) nitrate precursors, before HF activation. Figure S10: Isotropic lines of the ^19^F MAS experimental spectra of the MgF_2_ samples prepared from acetate, carbonate, chloride and nitrate precursors, before and after HF activation. Figure S11: ^19^F MAS experimental and fitted NMR spectra of the MgF_2_ samples prepared from acetate, carbonate, chloride and nitrate precursors, after HF activation. Figure S12: ^19^F MAS experimental and fitted NMR spectra of the MgF_2_ sample prepared from nitrate precursor, after HF activation. Figure S13: ^1^H MAS experimental and fitted NMR spectra of the MgF_2_ samples prepared from acetate, carbonate, chloride and nitrate precursors, before HF activation. Figure S14: ^1^H MAS experimental and fitted NMR spectra of the MgF_2_ samples prepared from acetate, carbonate, chloride and nitrate precursors, after HF activation. Figure S15: Thermogravimetric analysis (TGA) of as-synthesized MgF_2_ nanoparticles prepared from acetate, carbonate, chloride and nitrate magnesium precursors. Figure S16: Mass-spectrometry coupled thermogravimetric analysis (MS-TGA) of as-synthesized MgF_2_-acetate nanoparticles. Table S1: Results of Le Bail refinements of MgF_2_ nanoparticles prepared from acetate, carbonate, chloride and nitrate precursors. Table S2: Isotropic chemical shifts *δ*_iso_ (ppm), line widths LW (ppm), relative intensities I (%) and assignment of the NMR lines used for the fits of the ^19^F solid state MAS (64 kHz) NMR spectra of the MgF_2_ samples prepared from (a) acetate, (b) carbonate, (c) chloride and (d) nitrate precursors, before HF activation. Table S3: Isotropic chemical shifts *δ*_iso_ (ppm), line widths LW (ppm), relative intensities I (%) and assignment of the NMR lines used for the fits of the ^19^F solid state MAS (64 kHz) NMR spectra of the MgF_2_ samples prepared from (a) acetate, (b) carbonate, (c) chloride and (d) nitrate precursors, after HF activation. Additionally, for (d), relative intensities I’ (%) of the NMR lines assigned to MgF_2_. Table S4: F-Mg and F-F distances (Å) in MgF_2_ (W. H. Baur, Acta Crystallogr. B 32 (1976) 2200–2204). Table S5: Proportions (%) of the FMg_3_F_11−y_(OH)_y_ environments, as a function of the composition (x value) in MgF_2−x_(OH)_x_, assuming random distribution of OH− and F− ions at anionic sites. For these x values, all probabilities for y ≥ 5 are negligible (<0.05%). Table S6: Isotropic chemical shifts *δ*_iso_ (ppm), line widths LW (ppm), relative intensities I (%) and tentative assignment of the NMR lines used for the fits of the ^1^H solid state MAS (60 kHz) NMR spectra of the MgF_2_ samples prepared from (a) acetate, (b) carbonate, (c) chloride and (d) nitrate precursors, before HF activation. Table S7: Isotropic chemical shifts *δ*_iso_ (ppm), line widths LW (ppm), relative intensities I (%) and tentative assignment of the NMR lines used for the fits of the ^1^H solid state MAS (60 kHz) NMR spectra of the MgF_2_ samples prepared from (a) acetate, (b) carbonate, (c) chloride and (d) nitrate precursors, after HF activation.
