# Supplementary material for: The Effects of Various Parameters of the Microwave-Assisted Solvothermal Synthesis on the Specific Surface Area and Catalytic Performance of MgF2 Nanoparticles
Source: Materials (Basel). 2020 Aug 12;13(16):3566. doi: 10.3390/ma13163566 (PMC7475908; doi:10.3390/ma13163566)
Supplement: Supplementary file 1 [file materials-13-03566-s001.pdf]

materials
MDPI

Suplementary Materials

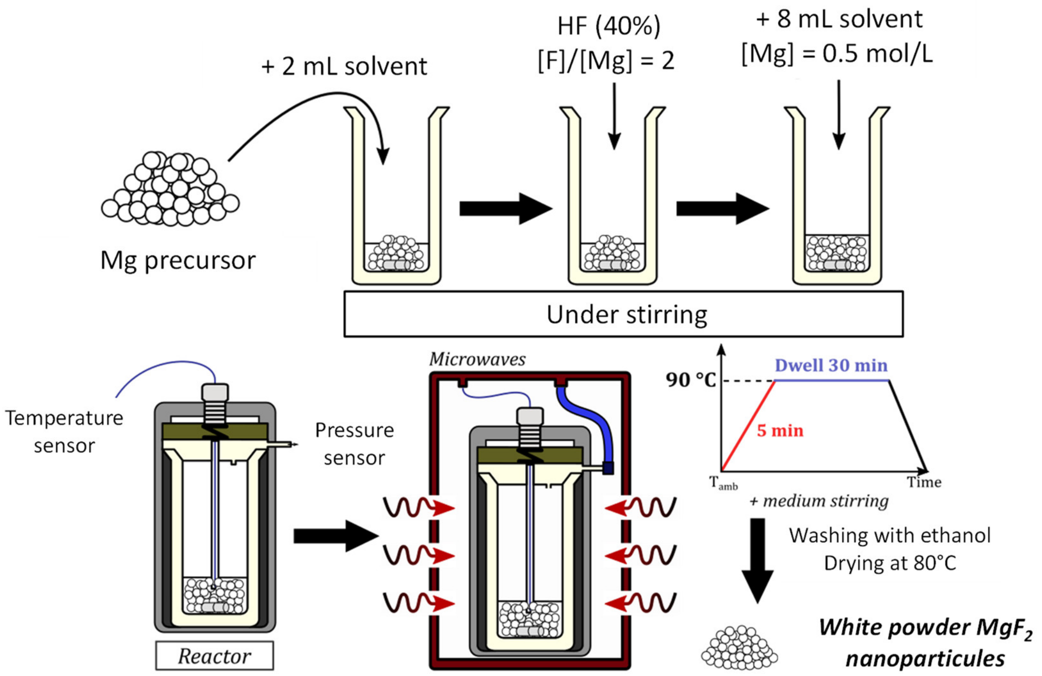

Figure S1. Representation of microwaves assisted synthesis of MgF_2 nanoparticles.

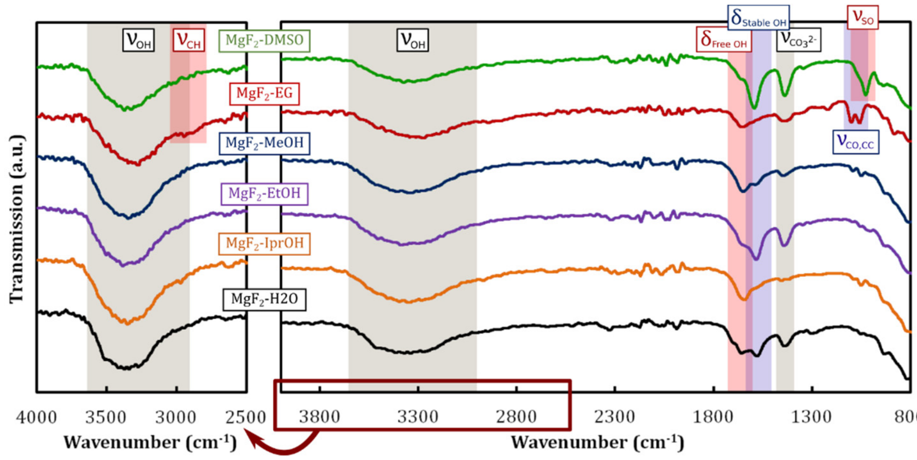

Figure S2. FT-IR spectra of MgF_2 powders synthetized using H_2O, ^{i}PrOH, EtOH, EG and DMSO as solvent.

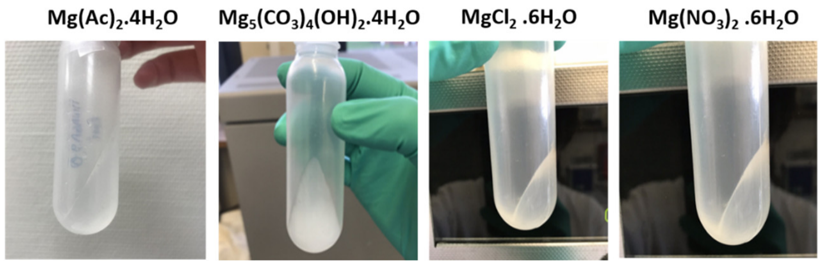

Figure S3. Pictures of the as-synthesized MgF_2 nanoparticles as gel after the washing procedure and before drying to obtain a white powder.

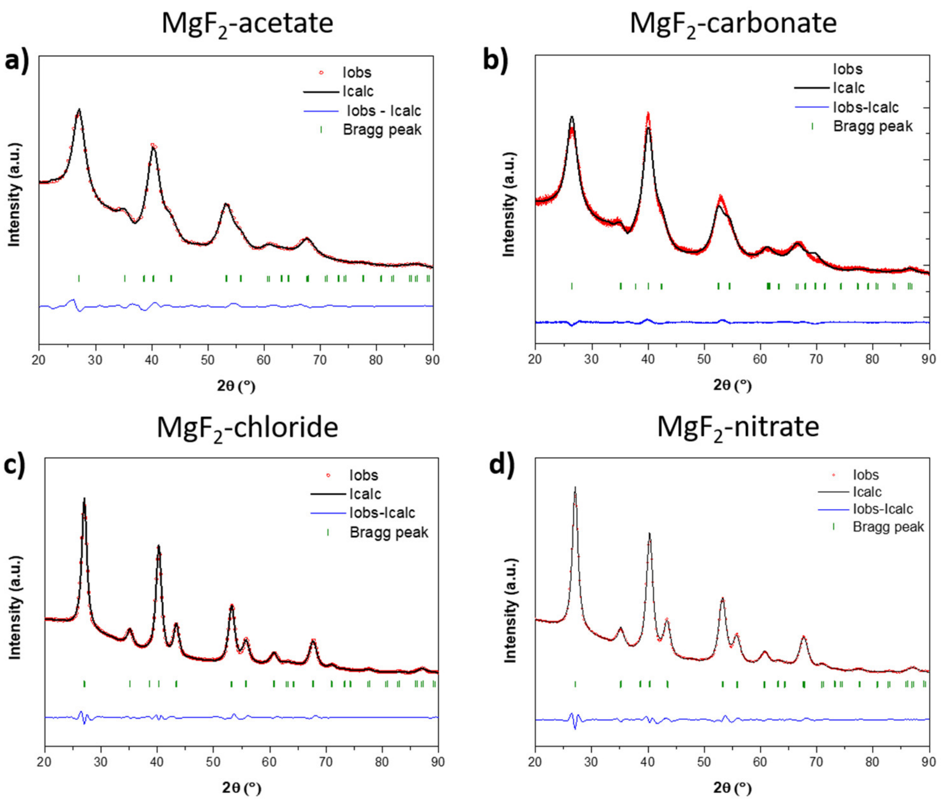

Figure S4: Le Bail refinements of MgF_2 nanoparticles prepared from (**a**) acetate, (**b**) carbonate, (**c**) chloride and (**d**) nitrate magnesium precursors. Vertical markers give the Bragg peak positions of the crystalline structure of MgF_2 (space group $P4_2/mnm$ (n°136)).

Table S1. Results of Le Bail refinements of MgF_2 nanoparticles prepared from nitrate precursor.

	**acetate**	**carbonate**	**chloride**	**nitrate**
a (Å)	4.670(2)	4.7150(7)	4.6526(4)	4.6544(6)
c (Å)	3.0248(9)	3.0004(4)	3.0458(2)	3.0476(3)
R_p, R_{wp}; χ^2	6.32%; 5.83%; 3.13%	4.72%; 5.99%;8.17%	4.68%; 5.56%;4.54%	5.25%,5.31%,7.74%
Integral-breadth apparent size <L> (nm)	3	3	6	5
Diameter of the spherical particles D_{XRD} (nm)	4	4	8	7

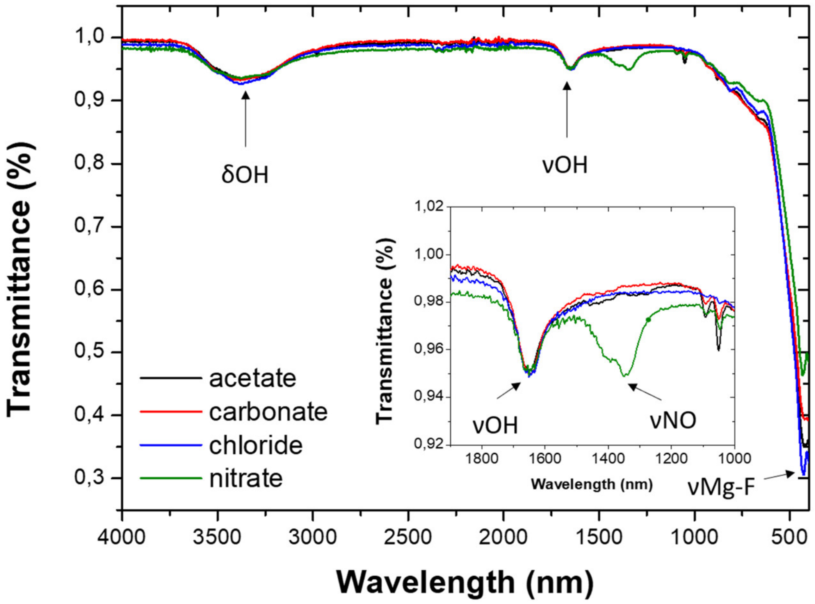

Figure S5. FT-IR spectra of MgF_2 nanoparticles prepared from acetate, carbonate, chloride, and nitrate magnesium precursors and using MeOH as solvent.

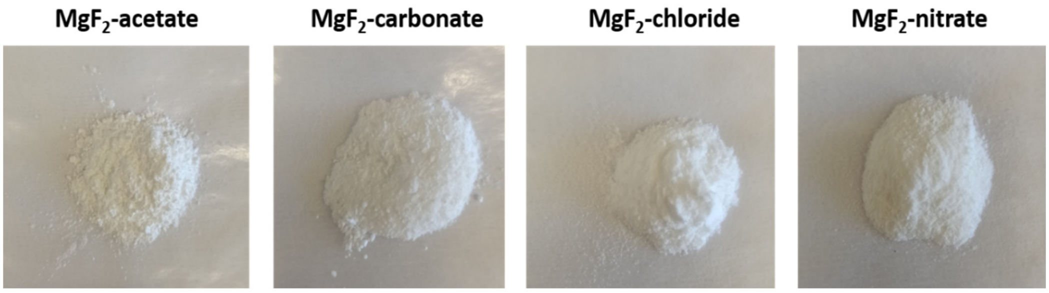

Figure S6. Pictures MgF_2 powders prepared from prepared from acetate, carbonate, chloride and nitrate precursors.

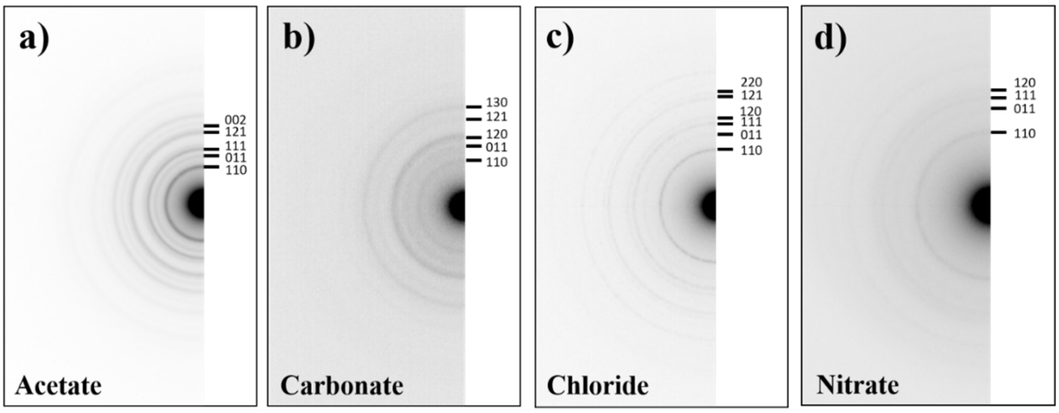

Figure S7. SAED patterns of MgF_2 nanoparticles prepared from (**a**) acetate, (**b**) carbonate, (**c**) chloride and (**d**) nitrate precursors.

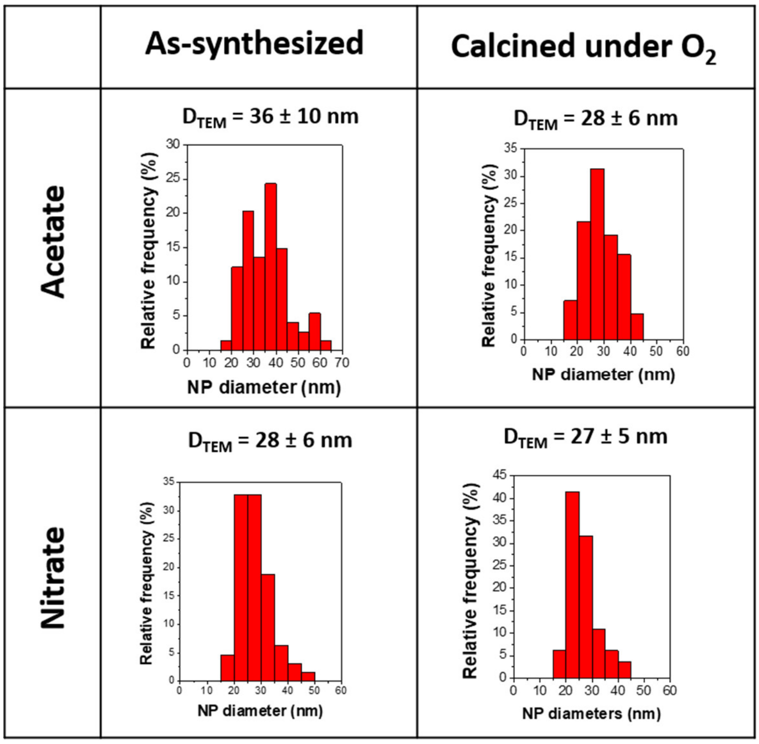

Figure S8. Nanoparticle sizes of MgF_2 after HF treatment measured from TEM images.

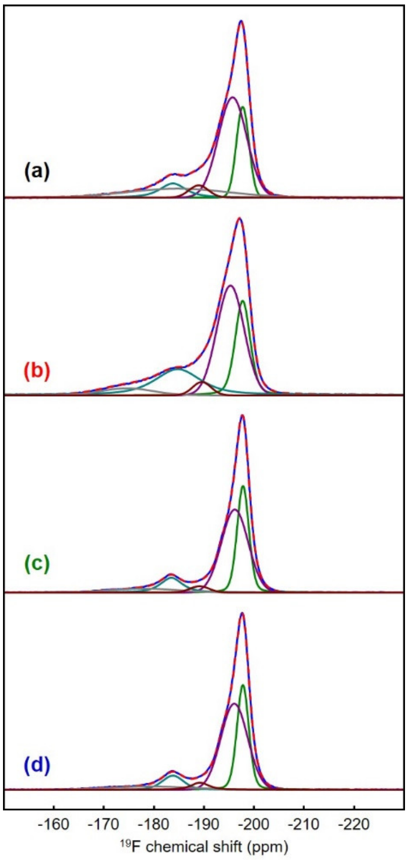

Figure S9. ^{19}F MAS (64 kHz) experimental (blue line) and fitted (dashed red line) NMR spectra of the MgF_2 samples prepared from (**a**) acetate, (**b**) carbonate, (**c**) chloride and (**d**) nitrate precursors, before HF activation. The individual resonances used for each fit are shown below each spectrum (see **Table S2**).

Table S2. Isotropic chemical shifts δ_{iso} (ppm), line widths LW (ppm), relative intensities I (%) and assignment of the NMR lines used for the fits of the ^{19}F solid state MAS (64 kHz) NMR spectra of the MgF_2 samples prepared from (a) acetate, (b) carbonate, (c) chloride and (d) nitrate precursors, before HF activation.

	δ_{iso}	LW	I	Assignment
	-197.8	3.0	20.2	FMg_3F_{11}
	-195.7	6.7	50.9	FMg_3F_{11} & $FMg_3F_{11-y}(OH)_y$ ($y \geq 1$)
**(a)**	-189.0	4.5	4.2	$FMg_3F_{11-y}(OH)_y$ ($y \geq 1$)
	-184.7	*23.8*	16.2	$FMg_3F_{11-y}(OH)_y$ ($y \geq 1$)
	-183.8	6.1	8.5	$FMg_3F_{11-y}(OH)_y$ ($y \geq 1$)
	-197.7	3.5	23.8	FMg_3F_{11}
	-195.3	6.4	45.0	FMg_3F_{11} & $FMg_3F_{11-y}(OH)_y$ ($y \geq 1$)
**(b)**	-189.6	4.3	3.7	$FMg_3F_{11-y}(OH)_y$ ($y \geq 1$)
	-184.8	10.7	21.9	$FMg_3F_{11-y}(OH)_y$ ($y \geq 1$)
	-174.6	12.2	5.6	$FMg_3F_{11-y}(OH)_y$ ($y \geq 1$)
	-197.8	2.8	31.2	FMg_3F_{11}
	-196.2	6.3	51.0	FMg_3F_{11} & $FMg_3F_{11-y}(OH)_y$ ($y \geq 1$)
**(c)**	-189.1	4.5	2.8	$FMg_3F_{11-y}(OH)_y$ ($y \geq 1$)
	-183.5	5.1	9.6	$FMg_3F_{11-y}(OH)_y$ ($y \geq 1$)
	-178.0	16.1	5.5	$FMg_3F_{11-y}(OH)_y$ ($y \geq 1$)
	-197.8	2.8	29.9	FMg_3F_{11}
	-196.1	6.3	52.0	FMg_3F_{11} & $FMg_3F_{11-y}(OH)_y$ ($y \geq 1$)
**(d)**	-189.2	4.2	3.7	$FMg_3F_{11-y}(OH)_y$ ($y \geq 1$)
	-183.8	5.1	7.8	$FMg_3F_{11-y}(OH)_y$ ($y \geq 1$)
	-179.6	19.0	6.7	$FMg_3F_{11-y}(OH)_y$ ($y \geq 1$)

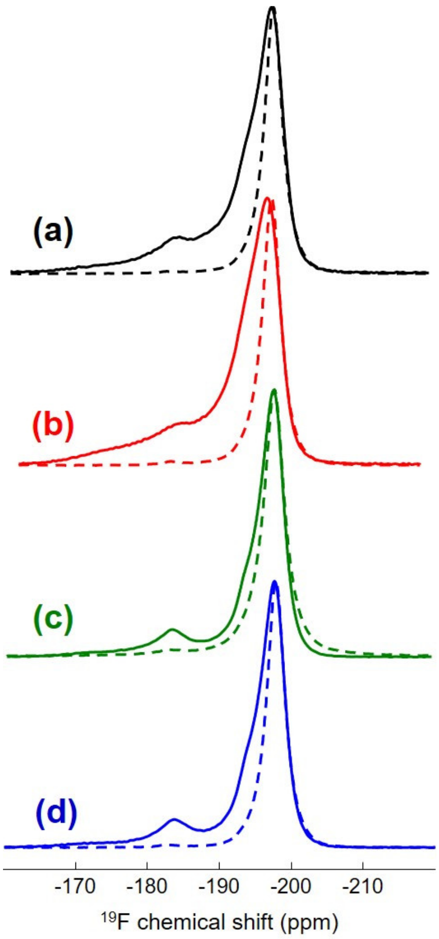

Figure S10. Isotropic lines of the ^{19}F MAS experimental spectra of the MgF_2 samples prepared from (**a**) acetate, (**b**) carbonate, (**c**) chloride and (**d**) nitrate precursors, before (solid lines) and after (dashed lines) HF activation.

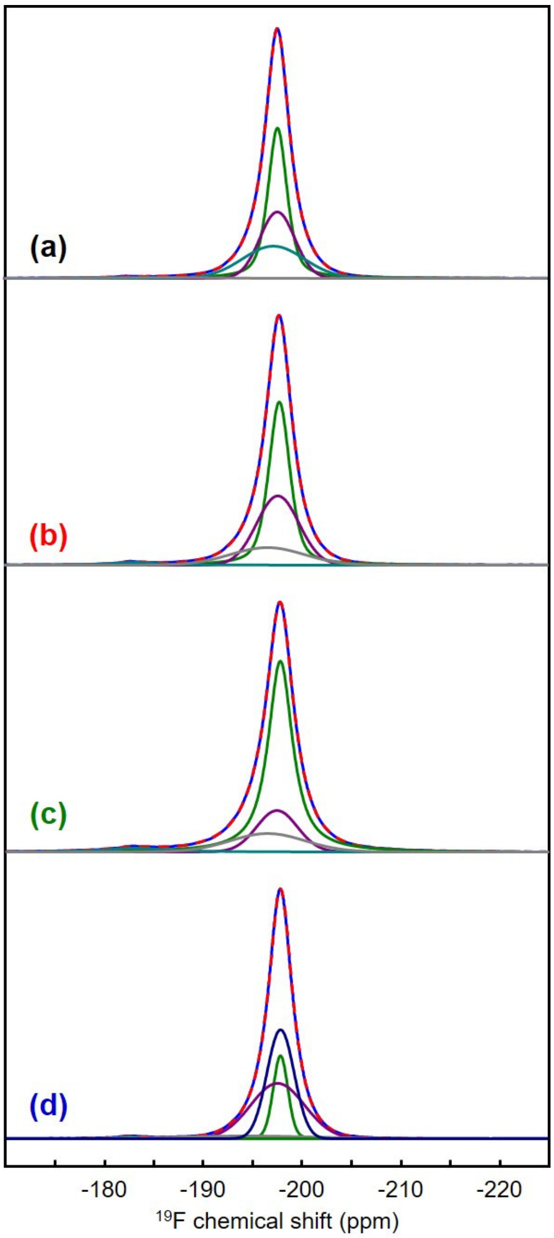

Figure S11. ^{19}F MAS (64 kHz) experimental (blue line) and fitted (dashed red line) NMR spectra of the MgF_2 samples prepared from (**a**) acetate, (**b**) carbonate, (**c**) chloride and (**d**) nitrate precursors, after HF activation. The individual resonances used for each fit are shown below each spectrum (see **Error! Reference source not found.**).

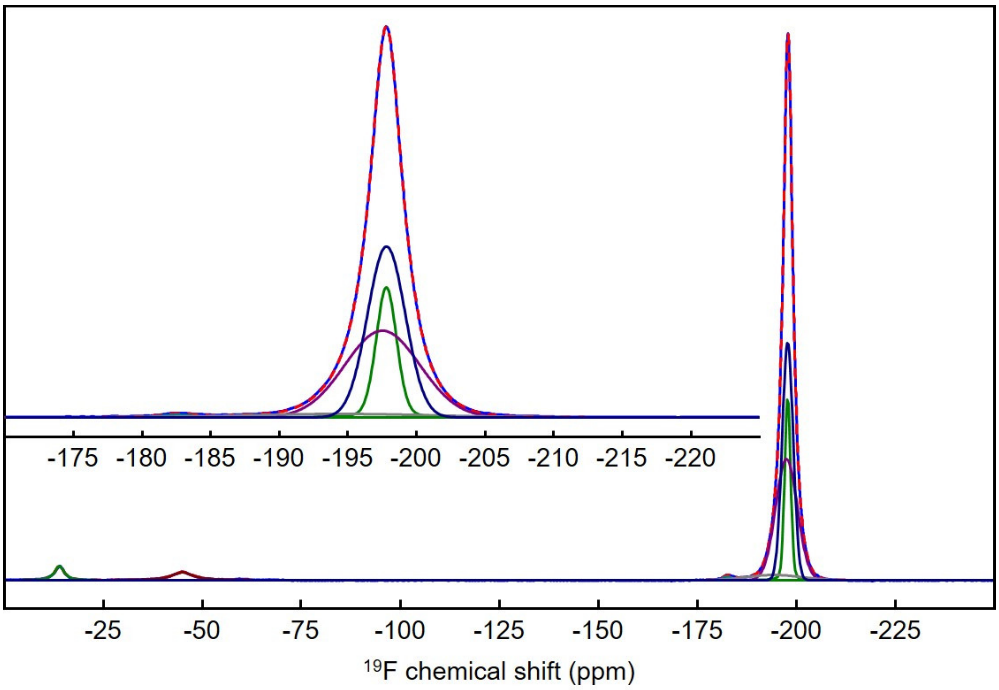

Figure S12. ^{19}F MAS (64 kHz) experimental (blue line) and fitted (dashed red line) NMR spectra of the MgF_2 sample prepared from nitrate precursor, after HF activation. The isotropic lines assigned to MgF_2 are expended in the inset. The individual resonances used for the fit are shown below (see **Error! Reference source not found.**).

Table S3. Isotropic chemical shifts δiso (ppm), line widths LW (ppm), relative intensities I (%) and assignment of the NMR lines used for the fits of the ^{19}F solid state MAS (64 kHz) NMR spectra of the MgF_2 samples prepared from (a) acetate, (b) carbonate, (c) chloride and (d) nitrate precursors, after HF activation. Additionally, for (d), relative intensities I′ (%) of the NMR lines assigned to MgF_2.

	δ_{iso}	LW	I	Assignment	I′
**(a)**	-197.71	2.3	43.4	FMg_3F_{11}	
	-197.69	4.2	28.7	FMg_3F_{11}	
	-197.3	7.4	26.0	FMg_3F_{11} & $FMg_3F_{11-y}(OH)_y$ ($y \geq 1$)	
	-185.6	11.3	2.0	$FMg_3F_{11-y}(OH)_y$ ($y \geq 1$)	
**(b)**	-197.85	2.5	47.9	FMg_3F_{11}	
	-197.70	5.0	35.0	FMg_3F_{11}	
	-196.6	8.8	14.9	FMg_3F_{11} & $FMg_3F_{11-y}(OH)_y$ ($y \geq 1$)	
	-183.2	4.9	2.2	$FMg_3F_{11-y}(OH)_y$ ($y \geq 1$)	
**(c)**	-197.8	3.0	64.0	FMg_3F_{11}	
	-197.5	4.9	17.2	FMg_3F_{11}	
	-196.5	8.9	14.8	FMg_3F_{11} & $FMg_3F_{11-y}(OH)_y$ ($y \geq 1$)	
	-183.2	7.6	4.0	$FMg_3F_{11-y}(OH)_y$ ($y \geq 1$)	
**(d)**	-197.8	3.2	36.0	FMg_3F_{11}	38.3
	-197.8	1.8	15.6	FMg_3F_{11}	16.6
	-197.5	6.4	37.5	FMg_3F_{11} & $FMg_3F_{11-y}(OH)_y$ ($y \geq 1$)	39.9
	-194.4	16.2	4.3	$FMg_3F_{11-y}(OH)_y$ ($y \geq 1$)	4.5
	-182.6	2.9	0.6	$FMg_3F_{11-y}(OH)_y$ ($y \geq 1$)	0.6
	-45.0	6.5	3.6	?	
	-13.9	2.9	2.6	? BaF_2 ?	

Table S4. F-Mg and F-F distances (Å) in MgF_2 (W. H. Baur, Acta Crystallogr. B 32 (1976) 2200–2204).

F1	Mg1	1x	1.984
	Mg1	2x	1.994
	F1	1x	2.576
	F1	8x	2.813
	F1	2x	3.045
	F1	2x	3.346

Table S5. Proportions (%) of the $FMg_3F1_{1-y}(OH)_y$ environments, as a function of the composition (x value) in $MgF_{2-x}(OH)_x$, assuming random distribution of OH^- and F^- ions at anionic sites. For these x values, all probabilities for y ≥ 5 are negligible (< 0.05%).

	**x**	**0.00**	**0.01**	**0.02**	**0.03**	**0.04**	**0.05**	**0.06**	**0.07**	**0.08**	**0.10**	**0.12**	**0.14**	**0.16**	**0.18**	**0.20**
	**2-x**	**2.00**	**1.99**	**1.98**	**1.97**	**1.96**	**1.95**	**1.94**	**1.93**	**1.92**	**1.9**	**1.88**	**1.86**	**1.84**	**1.82**	**1.80**
**y**	**11-y**	**Proportions**														
**0**	**11**	100.0	94.6	89.5	84.7	80.1	75.7	71.5	67.6	63.8	56.9	50.6	45.0	40.0	35.4	31.4
**1**	**10**	0.0	5.2	9.9	14.2	18.0	21.3	24.3	27.0	29.3	32.9	35.5	37.3	38.2	38.6	38.4
**2**	**9**	0.0	0.1	0.5	1.1	1.8	2.7	3.8	4.9	6.1	8.7	11.3	14.0	16.6	19.1	21.3
**3**	**8**	0.0	0.0	0.0	0.0	0.1	0.2	0.3	0.5	0.8	1.4	2.2	3.2	4.3	5.7	7.1
**4**	**7**	0.0	0.0	0.0	0.0	0.0	0.0	0.0	0.0	0.1	0.1	0.3	0.5	0.8	1.1	1.6
**5**	**6**	0.0	0.0	0.0	0.0	0.0	0.0	0.0	0.0	0.0	0.0	0.0	0.1	0.1	0.2	0.2

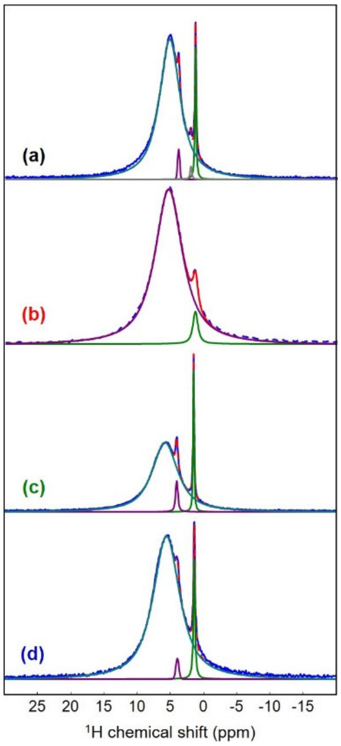

Figure S13. ^1H MAS (60 kHz) experimental (blue line) and fitted (dashed red line) NMR spectra of the MgF_2 samples prepared from (**a**) acetate, (**b**) carbonate, (**c**) chloride and (**d**) nitrate precursors, before HF activation. The individual resonances used for each fit are shown below each spectrum (see **Table S6**).

Table S6. Isotropic chemical shifts δ_{iso} (ppm), line widths LW (ppm), relative intensities I (%) and tentative assignment of the NMR lines used for the fits of the ^1H solid state MAS (60 kHz) NMR spectra of the MgF_2 samples prepared from (**a**) acetate, (**b**) carbonate, (**c**) chloride and (**d**) nitrate precursors, before HF activation.

	δ_{iso}	LW	I	Assignment
(a)	1.4	0.21	5.2	Mg_3-OH
	2.1	0.32	0.7	CH_3COOH
	3.9	0.38	1.5	absorbed H_2O molecules
	5.2	3.8	92.7	
(b)	1.4	0.9	3.7	Mg_3-OH
	5.4	5.0	96.3	absorbed H_2O molecules
(c)	1.4	0.17	7.5	Mg_3-OH
	3.9	0.42	3.6	absorbed H_2O molecules
	5.7	4.6	88.9	
(d)	1.4	0.28	5.0	Mg_3-OH
	3.9	0.54	1.2	absorbed H_2O molecules
	5.6	4.8	93.8	

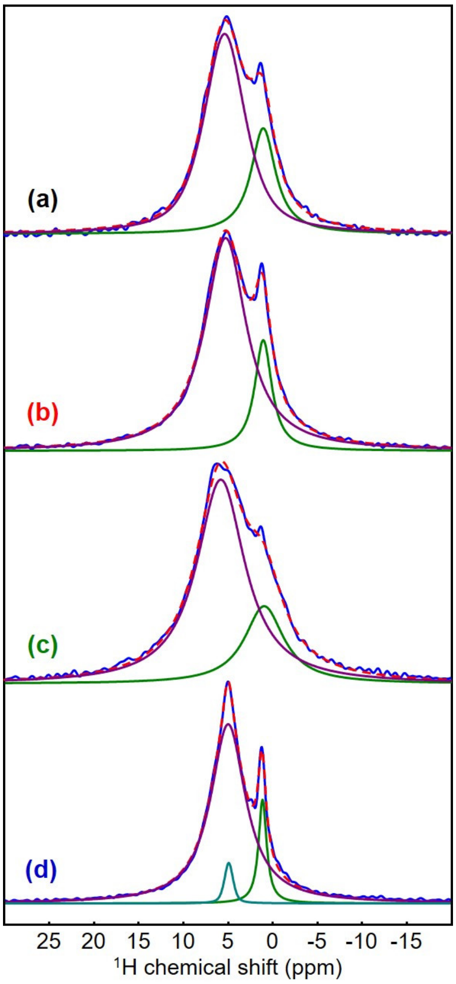

Figure S14. ^1H MAS (60 kHz) experimental (blue line) and fitted (dashed red line) NMR spectra of the MgF_2 samples prepared from (**a**) acetate, (**b**) carbonate, (**c**) chloride and (**d**) nitrate precursors, after HF activation. The individual resonances used for each fit are shown below each spectrum (see **Table S7**).

Table S7. Isotropic chemical shifts δ_{iso} (ppm), line widths LW (ppm), relative intensities I (%) and tentative assignment of the NMR lines used for the fits of the ^{1}H solid state MAS (60 kHz) NMR spectra of the MgF_2 samples prepared from (**a**) acetate, (**b**) carbonate, (**c**) chloride and (**d**) nitrate precursors, after HF activation.

	δ_{iso}	LW	I	Assignment
**(a)**	1.1	3.3	26.5	Mg_3-OH
	5.4	5.5	73.5	absorbed H_2O molecules
**(b)**	1.1	2.3	18.0	Mg_3-OH
	5.3	5.6	82.0	absorbed H_2O molecules
**(c)**	1.0	5.3	23.8	Mg_3-OH
	5.8	6.5	76.2	absorbed H_2O molecules
**(d)**	1.2	1.1	11.7	Mg_3-OH
	4.9	1.2	4.1	absorbed H_2O molecules
	5.0	4.8	84.3	

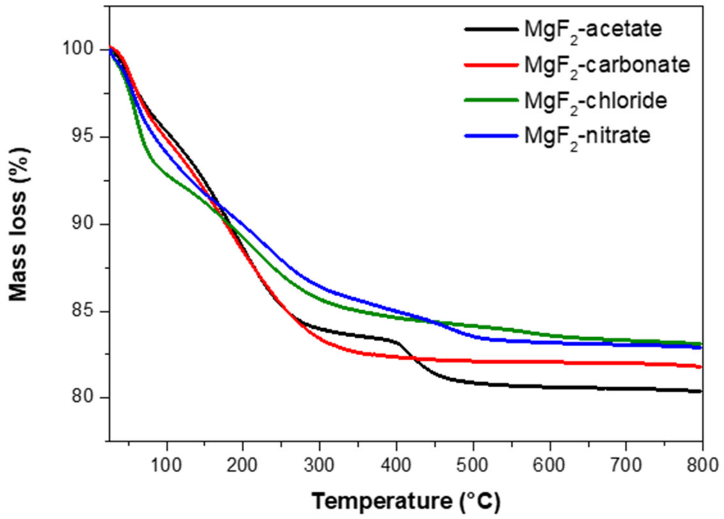

Figure S15. Thermogravimetric analysis (TGA) of as-synthesized MgF_2 nanoparticles prepared from acetate, carbonate, chloride and nitrate magnesium precursors.

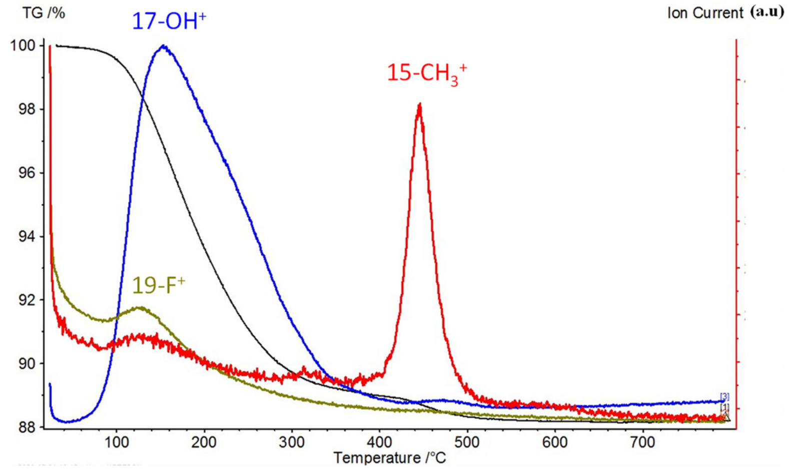

Figure S16. Mass-spectrometry coupled thermogravimetric analysis (MS-TGA) of as-synthesized MgF_2-acetate nanoparticles.

© 2020 by the authors. Submitted for possible open access publication under the terms and conditions of the Creative Commons Attribution (CC BY) license (http://creativecommons.org/licenses/by/4.0/).
